# Correlation of *Chitinase 3-Like 1* Single Nucleotide Polymorphisms and Haplotypes with Uterine Cervical Cancer in Taiwanese Women

**DOI:** 10.1371/journal.pone.0104038

**Published:** 2014-09-09

**Authors:** Yue-Shan Lin, Yu-Fan Liu, Ying-Erh Chou, Shun-Fa Yang, Ming-Hsien Chien, Chih-Hsien Wu, Chi-Hung Chou, Chao-Wen Cheng, Po-Hui Wang

**Affiliations:** 1 Department of Obstetrics and Gynecology, Chi-Mei Foundation Medical Center, Tainan, Taiwan; 2 Department of Biomedical Sciences, Chung Shan Medical University, Taichung, Taiwan; 3 Institute of Medicine, Chung Shan Medical University, Taichung, Taiwan; 4 Department of Medical Research, Chung Shan Medical University Hospital, Taichung, Taiwan; 5 Graduate Institute of Clinical Medicine, College of Medicine, Taipei Medical University, Taipei, Taiwan; 6 Wan Fang Hospital, Taipei Medical University, Taipei, Taiwan; 7 Division of Cardiology, Department of Internal Medicine, Yuan-Sheng Hospital and Changhua Christian Hospital, Yuanlin Branch, Yuanlin, Taiwan; 8 School of Medicine, Chung Shan Medical University, Taichung, Taiwan; 9 Department of Obstetrics and Gynecology, Chung Shan Medical University Hospital, Taichung, Taiwan; Georgetown University, United States of America

## Abstract

**Background:**

This study aimed to investigate the relationships of *chitinase 3-like 1* (*CHI3L1*) single nucleotide polymorphisms (SNPs) and haplotypes with the development of uterine cervical cancer in Taiwanese women. The SNPs frequencies and haplotypes were also correlated with the clinicopathologic variables of cervical cancer, cancer recurrence, and patient survival.

**Methodology and Principal Findings:**

Ninety-nine patients with invasive cancer and 61 with pre-cancerous lesions of the uterine cervix were compared to 310 healthy control subjects. Three SNPs rs6691378 (−1371, G/A), rs10399805 (−247, G/A) and rs4950928 (−131, C/G) in the promoter region, and one SNP rs880633 (+2950, T/C) in exon 5 were analyzed by real time polymerase chain reaction and genotyping. The results showed that the mutant homozygous genotype AA of *CHI3L1* SNP rs6691378 and AA of rs10399805, and haplotypes AACC and AACT increased the risk of developing pre-cancerous lesions and invasive cancer. The patients with these risk haplotypes had higher than stage I tumors, larger tumors, and vaginal invasion. In logistic regression model, they also tended to have poor survival event [*p* = 0.078; odds ratio (OR): 2.99, 95% confidence interval (CI): 0.89–10.08] and a higher probability of recurrence event (*p* = 0.081; OR: 3.07, 95% CI: 0.87–10.81). There was a significant association between the *CHI3L1* risk haplotypes and probability of recurrence (*p* = 0.002; hazard ratio: 6.21, 95% CI: 1.90–20.41), and a marginal association between the risk haplotypes and overall survival (*p* = 0.051; hazard ratio: 3.76, 95% CI: 0.99–14.29) in the patients with SCC, using Cox proportional hazard model.

**Conclusion:**

The *CHI3L1* SNPs rs6691378 and rs10399805 and *CHI3L1* haplotypes all correlated with the development of cervical pre-cancerous lesions and invasive cancer. The cervical cancer patients with the *CHI3L1* haplotypes AACC or AACT had poor clinicopathologic characteristics and poor recurrence and survival events. These risk haplotypes were associated with higher recurrence, especially in the patients with SCC.

## Introduction

Chitinase3-like1 (CHI3L1) is a glycoprotein encoded by the *chitinase 3-like 1* (*CHI3L1*) gene located on human chromosome 1q32.1 [1]. This glycoprotein is often referred to as YKL-40 or human cartilage glycoprotein-39 (HC gp-39) and is known to be a pro-inflammatory cytokine of chitinase [2], [3]. It is a secreted protein with a molecular weight of 40 kD and is identified by the N-terminal sequencing to be tyrosine (abbreviated as Y), lysine (K), and leucine (L) [4]. It is recognized as a growth factor for connective cells and a migration-promoting factor for endothelial cells, and is produced by a variety of cells including cancer cells, activated macrophages, and neutrophils. It is also known to play a role in inflammation, cell proliferation, anti-apoptosis, stimulation of angiogenesis, and regulation of extracellular tissue remodeling [2], [5]–[7].

Kjaergaard et al. used a population-based prospective study of the Danish general population to investigate the genetic variants of *CHI3L1* that influence YKL-40 levels, and found that eight single nucleotide polymorphisms (SNPs) of the *CHI3L1* gene are associated with plasma YKL-40 levels in the general population [8]. These *CHI3L1* SNPs included rs10399805 (−247, G/A) and rs4950928 (−131, C/G) in the promoter region, and rs880633 (+2950, T/C) in exon 5 etc. However, Thomsen et al., also using the Danish population in the international MONICA (MONItoring trends and determinants of CArdiovascular disease) project, demonstrated that 12SNPs were associated with serum YKL-40 levels [9]. These *CHI3L1* SNPs included rs6691378 (−1371, G/A), rs4950928, and rs880633 etc.

Uterine cervical cancer is the fifth most common type of malignancy among women in Taiwan, with an age-standardized incidence rate in 2009 of 11.86 per 100000 women according to the Health Promotion Administration of the Ministry of Health and Welfare. Its age-standardized mortality rate was 3.72 per 100000 women in 2011, making it the seventh top cause of cancer death in Taiwanese women.

Single nucleotide polymorphisms may affect promoter activity, gene expression, messenger RNA conformation (stability), and sub-cellular localization of mRNAs and/or proteins, and probably cause diseases [10]. Several recent studies have shown that genetic variations of *CHI3L1* SNPs have an impact on inter-individual serum YKL-40 levels as well as susceptibilities to atopy, sarcoidosis, asthma, and lung function [11]–[14]. Pre-treatment serum levels of YKL-40 have also been reported to be elevated in cervical cancer [15]. To date, no study has correlated *CHI3L1* SNPs with cervical cancer in Taiwanese women. Under the hypothesis that gene polymorphisms or haplotypes of the *CHI3L1* gene have an impact on YKL-40 expression in cervical cancer, this study investigated the distribution of *CHI3L1* gene polymorphisms and haplotypes among patients with cervical cancer and pre-cancerous lesions and normal controls to define their roles in cervical carcinogenesis in Taiwanese women. The SNP frequencies or haplotypes of *CHI3L1* were further associated with clinicopathologic variables of cervical cancer, cancer recurrence, and patient survival. This study demonstrated significant associations of *CHI3L1* SNPs and haplotypes with the development of pre-cancerous lesions and invasive cancer of the uterine cervix, and revealed that *CHI3L1* haplotypes were related to the prognosis of cervical cancer patients.

## Materials and Methods

### Population

Four hundred and seventy women, including 99 patients with invasive cancer, 61 patients with pre-cancerous lesions of the uterine cervix, and 310 normal controls, were recruited consecutively into this study. The patients with invasive cervical cancer were clinically staged based on the 2009 International Federation of Gynecology and Obstetrics Classification and received routine treatment protocols at the Department of Obstetrics and Gynecology in Chung Shan Medical University Hospital, Taiwan, from March 1999 to October 2012. The patients with pre-cancerous lesions, which only comprised cervical high-grade dysplasia (high-grade squamous intraepithelial lesions) in this study and included moderate and severe dysplasia as well as carcinoma in situ, underwent colposcopy-directed cervical punch biopsy, large loop excision of the transformation zone, total abdominal hysterectomy, or total vaginal hysterectomy. The 310 controls with normal Papanicolaou smears were further verified using colposcopy during general examinations at the outpatient department of our hospital. The ages of the women with cervical invasive cancer, pre-cancerous lesions, and normal controls were 53.6±12.0, 42.7±12.7, and 44.7±9.8(mean ± SD) years, respectively. All of them were Taiwanese women who resided in central Taiwan. The Chung Shan Medical University Hospital Institutional Review Board approved this study (CSMUH IRB: CS12218, CS12219, CS14014), and informed written consent was obtained from each individual.

### Selection of *chitinase 3-like 1* gene polymorphisms

Three SNPs rs6691378 (−1371, G/A), rs10399805 (−247, G/A), and rs4950928 (−131, C/G) in the promoter region and one SNP rs880633 (+2950, T/C) in exon 5 were selected based on the Chinese HapMap (Han Chinese in Beijing, China) data and the studies of Thomsen et al. and Kjaergaard et al. [12], [16]. The minor allele frequencies (MAFs) of these SNPs were ≥5%.

### Blood sample collection and genomic DNA extraction

In total, 160 blood specimens were collected from the patients with cervical invasive cancer and those with pre-cancerous lesions, and 310 blood specimens were obtained from the controls. Genomic DNA was extracted from EDTA anti-coagulated venous blood using a QIAamp DNA blood mini kit (Qiagen,Valencia, CA, USA) based on the manufacturer's protocol. The DNA was dissolved in Tris ethylene buffer (10 mmol/L Tris and 1 mmol/L EDTA; pH 7.8) and then quantified by a measurement of OD260. The final preparation was stored at −20°C and applied as templates in polymerase chain reaction (PCR).

### Single nucleotide polymorphisms by real time-PCR and genotyping

Allelic discrimination of the rs6691378 (−1371, G/A), rs10399805 (−247, G/A), rs4950928 (−131, C/G), and rs880633 (+2950, T/C) polymorphisms was assessed using an ABI StepOne Real-Time PCR System (Applied Biosystems, Foster City, CA, USA), and analyzed by SDS version 3.0 software (Applied Biosystems) using the TaqMan assay. The 10 µL final volume for each reaction contained 5 µL TaqMan Genotyping Master Mix, 0.25 µL TaqMan probe mix, and 10 ng genomic DNA. Real-time PCR included an initial denaturation step at 95°C for 10 minutes, followed by 40 cycles of 95°C for 15 seconds and then 60°C for one minute.

### Statistical analysis

Analysis of variance (ANOVA) was used to analyze the age distribution of the study population and control subjects. Scheffe's test was used for post hoc analysis.

Hardy-Weinberg equilibrium was used to analyze the genotype distributions of rs6691378, rs10399805, rs4950928, and rs880633 in the normal controls (degree of freedom = 2). Chi-square or Fisher exact tests were used to examine the relationships among frequencies of *CHI3L1* gene SNPs, alleles, and haplotypes, and the incidence of cervical neoplasia (including invasive cancer and pre-cancerous lesions), as well as various clinicopathologic parameters, including clinical stage (I or ≥II), histopathologic types such as squamous cell carcinoma (SCC) or adenocarcinoma, cell grading (well, or moderate and poor differentiation), invasion depth of cervical stroma (≤10 mm or >10 mm of stromal invasion depth), tumor diameter (≤4 or >4 cm), parametrium and vaginal invasion, and pelvic lymph node metastasis found during surgery.

Logistic regression model or multiple logistic regression were used to analyze multiple comparisons of SNP genotypes of the *CHI3L1* gene polymorphisms before and after controlling for age between the patients with cervical neoplasia and the controls, or among patients with invasive cancer or pre-cancerous lesions and the controls. No age distribution was adjusted for allele comparisons (the allele number of each allele in each subgroup equaled two-fold of the number of homozygous genotypes plus one-fold of the number of heterozygous genotypes, therefore we could not analyze the age distribution among the subjects subgroups; for example the number of G alleles = 2 * the number of GG alleles+1 * the number of GA alleles) or haplotype distribution (arbitrary reclassification). The sample size was estimated with power (1-β), 0.8 and α, 0.05, and the powers were calculated when the comparisons among the subgroups reached statistical significances (*p*<0.05) with an adequate sample size using WinPepi software, version 10.0.

A logistic regression model was also used to correlate the various clinicopathologic parameters and *CHI3L1* polymorphisms with cancer recurrence event or patient survival event. A Cox proportional hazard model was used to evaluate the effects of *CHI3L1* haplotypes on the probability of recurrence or overall survival after adjusting for various clinicopathologic parameters in multivariate analysis relative to recurrence or survival time. When the follow-up period was included into survival or recurrence analysis, the patients were enrolled for overall survival analysis including 5-year survival rate or probability of recurrence between primary surgery and death or recurrence or the end of the study (October, 2013) using the Kaplan-Meier model and multivariate and univariate Cox regression models. A significant difference was set at *p*<0.05. Statistical analyses including odds ratio (OR) and adjusted odds ratio (AOR; controlling for age) and their 95% confidence interval (CI) were calculated by the SPSS, version 12.0 and WinPepi Software, version 10.0.

## Results

The age distribution of the study subjects was significantly different between the patients with cervical cancer and those with pre-cancerous lesions (53.6±12.0 vs. 42.7±12.7 years, *p*<0.001) and between those with cervical cancer and the controls (53.6±12.0 vs. 44.7±9.8 years, *p*<0.001), but not between those with pre-cancerous lesions and the controls (42.7±12.7 vs. 44.7±9.8 years, *p* = 0.407). The genotype distributions of rs6691378, rs10399805, rs4950928, and rs880633 met the Hardy-Weinberg equilibrium, which was applied to the normal controls.

### Association of *chitinase 3-like 1* gene polymorphisms with cervical neoplasia

There were significant differences in the distributions of SNPs rs6691378 and rs10399805 of the *CHI3L1* gene between the women with cervical neoplasia and the normal controls (*p*<0.001 and *p*<0.001, respectively) (Table 1). However, no such differences were observed in rs4950928 and rs880633. The mutant homozygous genotype AA of SNP rs6691378 was differently distributed between women with cervical neoplasia and the controls compared to the wild homozygous genotype GG and heterozygous genotype GA (*p*<0.001). Further controlling for age, the women with the mutant homozygous AA carried a higher risk of developing cervical neoplasia compared to those with the wild genotype GG (AOR: 3.53, 95% CI: 1.71–7.35) or GG/GA (AOR: 3.40, 95% CI: 1.69–6.85) (Table 1). The mutant homozygous genotype AA of SNP rs10399805 was also differently distributed between those with cervical neoplasia and the controls compared to the wild GG and heterozygous GA (*p*<0.001). After controlling for age, the women with the mutant homozygous AA had a higher risk of developing cervical neoplasia compared to those with the wild genotype GG (AOR: 3.68, 95% CI: 1.71–7.87) or GG/GA (AOR: 3.60, 95% CI: 1.82–7.52).

**Table 1:** Genotype distributions of the single nucleotide polymorphisms of the *chitinase 3-like 1* gene in patients with neoplasia of the uterine cervix and normal controls.

Variables	Normal controls (n = 310)	Cervical neoplasia^b^ (n = 160)	*P* (power)^a^	Odds ratio (95% confidence interval)	Adjusted odds ratio (95% confidence interval)^c^
**rs6691378**					
GG^d^	156(50.3%)	67 (41.9%)	<0.001^a^	1.00	1.00
GA	138 (44.5%)	67 (41.9%)	(1.0)	1.13 (0.75–1.70)	1.08 (0.71–1.64)
AA	16 (5.2%)	26 (16.2%)		3.79 (1.90–7.52)	3.53 (1.71–7.35)
GG^d^	156 (50.3%)	67 (41.9%)	0.082	1.00	1.00
GA/AA	154 (49.7%)	93 (58.1%)		1.41(0.96–2.07)	1.32 (0.89–1.96)
GG/GA^d^	294 (94.8%)	134 (83.2%)	<0.001^a^	1.00	1.00
AA	16 (5.2%)	26 (16.3%)	(1.0)	3.57 (1.85–6.85)	3.40 (1.69–6.85)
rs10399805					
GG^d^	166 (53.5%)	73 (45.6%)	<0.001^a^	1.00	1.00
GA	130 (42.0%)	63 (39.4%)	(1.0)	1.10 (0.73–1.66)	1.05 (0.69–1.59)
AA	14 (4.5%)	24 (15.0%)		3.89 (1.91–7.94)	3.68 (1.71–7.87)
GG^d^	166(53.5%)	73 (45.6%)	0.103	1.00	1.00
GA/AA	144(46.5%)	87 (54.4%)		1.37 (0.94–2.02)	1.28 (0.87–1.90)
GG/GA^d^	296 (95.5%)	136 (85.0%)	<0.001^a^	1.00	1.00
AA	14 (4.5%)	24 (15.0%)	(1.0)	3.73 (1.87–7.46)	3.60 (1.82–7.52)
rs4950928					
CC^d^	204 (65.8%)	119 (74.4%)	0.162	1.00	1.00
CG	100 (32.3%)	39 (24.4%)		0.67 (0.43–1.03)	0.75 (0.48–1.16)
GG	6 (1.9%)	2 (1.2%)		0.57 (0.11–2.87)	0.48 (0.09–2.46)
CC^d^	204 (65.8%)	119 (74.4%)	0.058	1.00	1.00
CG/GG	106 (34.2%)	41 (25.6%)		0.66 (0.43–1.02)	0.73 (0.47–1.12)
CC/CG^d^	304 (98.1%)	158 (98.8%)	0.586	1.00	1.00
GG	6 (1.9%)	2 (1.2%)		0.64 (0.13–3.22)	0.52 (0.10–2.65)
rs880633					
TT^d^	131 (42.3%)	75(46.9%)	0.606	1.00	1.00
TC	148 (47.7%)	69(43.1%)		0.81 (0.54–1.22)	0.78 (0.52–1.18)
CC	31 (10.0%)	16 (10.0%)		0.90 (0.46–1.76)	0.90 (0.46–1.79)
TT^d^	131 (42.3%)	75(46.9%)	0.339	1.00	1.00
TC/CC	179 (57.7%)	85 (53.1%)		0.83 (0.57–1.22)	0.80 (0.54–1.18)
TT/TC^d^	279 (90.0%)	144 (90.0%)	1.00	1.00	1.00
CC	31 (10.0%)	16 (10.0%)		1.00 (0.53–1.89)	1.02 (0.53–1.97)

Statistical analysis: logistic regression model, Chi-square or Fisher exact tests.

a *p*<0.05; the sample size was estimated with power (1-β), 0.8 and α, 0.05 and the powers were calculated when the comparisons among the subgroups reached statistical significances (*p*<0.05) with an adequate sample size using WinPepi software, version 10.0.

b Cervical neoplasia included pre-cancerous lesions and invasive cancer of the uterine cervix.

c The adjusted odds ratios and their 95% confident intervals were estimated by logistic regression model after controlling for age.

d Used as a reference for comparisons to evaluate the odds ratio of other genotypes.

When the cervical neoplasia group was further subdivided into subgroups of invasive cancer and pre-cancerous lesions, significant differences existed in the distributions of SNP rs6691378 and rs10399805 of the *CHI3L1* gene among the women with cervical invasive cancer and pre-cancerous lesions and the normal women (*p* = 0.002 and *p* = 0.003, respectively; Table2). However, no such differences were observed in rs4950928 and rs880633. The mutant homozygous genotypes of both SNPs exhibited different distributions among the patients with cervical invasive cancer and pre-cancerous lesions, and the controls compared to the wild homozygous and heterozygous genotypes (*p*<0.001 and *p*<0.001, respectively; Table 2). After adjusting for age, the mutant homozygous AA in both SNPs rs6691378 and rs10399805 not only increased the risk of developing invasive cervical cancer (AOR: 2.37, 95% CI: 1.04–5.38 and AOR: 2.82, 95% CI: 1.20–6.58, respectively) but also pre-cancerous lesions (AOR: 5.26, 95% CI: 2.22–12.50 and AOR: 4.83, 95% CI: 1.90–12.35, respectively) compared to GG/GA (Table 2).

**Table 2:** Genotype distributions of the single nucleotide polymorphisms of *chitinase 3-like 1* gene in the patients with invasive cancer and pre-cancerous lesions of the uterine cervix and normal controls.

Variables	Normal controls (n = 310)	Pre-cancerous lesions (n = 61)	Invasive cancer(n = 99)	*P* (power)^a^	AOR(95% CI)^b^	AOR (95% CI)^c^
rs6691378						
GG^d^	156 (50.3%)	25 (41.0%)	42 (42.4%)	0.002^a^	1.00	1.00
GA	138 (44.5%)	25 (41.0%)	42 (42.4%)	(0.9)	1.14 (0.62–.08)	1.01 (0.60–1.69)
AA	16 (5.2%)	11 (18.0%)	15 (15.2%)		5.62 (2.24–14.08)	2.38 (1.01–5.62)
GG^d^	156 (50.3%)	25 (41.0%)	42 (42.4%)	0.217	1.00	1.00
GA/AA	154 (49.7%)	36 (59.0%)	57 (57.6%)		1.51 (0.86–2.64)	1.15 (0.71–1.88)
GG/GA^d^	294 (94.8%)	50 (82.0%)	84 (84.8%)	<0.001^a^	1.00	1.00
AA	16 (5.2%)	11 (18.0%)	15 (15.2%)	(1.0)	5.26 (2.22–12.50)	2.37(1.04–5.38)
rs10399805						
GG^d^	166 (53.5%)	29 (47.5%)	44 (44.4%)	0.003^a^	1.00	1.00
GA	130 (42.0%)	23 (37.7%)	40 (40.4%)	(0.9)	1.02 (0.56–1.86)	1.02 (0.61–1.71)
AA	14 (4.5%)	9 (14.8%)	15 (15.2%)		4.88 (1.85–12.82)	2.85 (1.18–6.90)
GG^d^	166 (53.5%)	29 (47.5%)	44 (44.4%)	0.247	1.00	1.00
GA/AA	144 (46.5%)	32 (52.5%)	55 (55.6%)		1.32 (0.76–2.29)	1.20 (0.74–1.96)
GG/GA^d^	296 (95.5%)	52 (85.2%)	84(84.8%)	<0.001^a^	1.00	1.00
AA	14 (4.5%)	9 (14.8%)	15(15.2%)	(1.0)	4.83 (1.90–12.35)	2.82 (1.20–6.58)
rs4950928						
CC^d^	204 (65.8%)	43 (70.5%)	76 (76.8%)	0.361	1.00	1.00
CG	100 (32.3%)	17 (27.9%)	22 (22.2%)		0.74 (0.40–1.37)	0.72 (0.41–1.27)
GG	6 (1.9%)	1(1.6%)	1 (1.0%)		0.89 (0.10–7.63)	0.35 (0.04–3.02)
CC^d^	204(65.8%)	43 (70.5%)	76 (76.8%)	0.117	1.00	1.00
CG/GG	106(34.2%)	18 (29.5%)	23 (23.2%)		0.75 (0.41–1.37)	0.69 (0.40–1.20)
CC/CG^d^	304 (98.1%)	60(98.4%)	98 (99.0%)	0.825	1.00	1.00
GG	6 (1.9%)	1(1.6%)	1 (1.0%)		0.96 (0.11–8.20)	0.38 (0.04–3.27)
rs880633						
TT^d^	131 (42.3%)	34 (55.7%)	41 (41.4%)	0.335	1.00	1.00
TC	148 (47.7%)	23 (37.7%)	46 (46.5%)		0.60 (0.33–1.06)	0.96 (0.58–1.61)
CC	31 (10.0%)	4 (6.6%)	12 (12.1%)		0.50 (0.17–1.52)	1.31 (0.58–2.96)
TT^d^	131 (42.3%)	34(55.7%)	41(41.4%)	0.131	1.00	1.00
TC/CC	179 (57.7%)	27 (44.3%)	58(58.6%)		0.58 (0.33–1.01)	1.02 (0. 63–1.66)
TT/TC^d^	279(90.0%)	57(93.4%)	87 (87.9%)	0.523	1.00	1.00
CC	31 (10.0%)	4 (6.6%)	12 (12.1%)		0.64 (0.22–1.89)	1.34 (0.62–2.88)

Statistical analysis: multiple logistic regression or Chi-square or Fisher exact tests. The AORs with their 95% CIs were estimated by the multiple logistic regression model after controlling for age.

a *p*<0.05; the sample size was estimated with power (1-β), 0.8 and α, 0.05 and the powers were calculated when the comparisons among the subgroups reached astatistical significances (*p*<0.05) with an adequate sample size using WinPepi software, version 10.0.

b Comparison between patients with pre-cancerous cervical lesions and normal controls after adjustments for age.

c Comparison between patients with cervical cancer and normal controls after adjustments for age.

d Used as a references for comparisons to evaluate the odds ratio of other genotypes.

Abbreviations: AOR, adjusted odds ratio; 95% CI, 95% confidence interval.

### Analysis of allele frequencies of *chitinase 3-like 1* polymorphisms in the study cohort

Analyzing the allele frequencies of the four *CHI3L1* gene polymorphisms in the 470 samples collected, the allele frequencies in *CHI3L1* SNPs rs6691378 and rs10399805 polymorphisms were differently distributed among the women with cervical invasive cancer, those with pre-cancerous lesions, and the normal subjects (*p* = 0.008 and *p* = 0.012, respectively) (Table 3). There were no such differences in SNPs rs4950928 and rs880633. The mutant alleles A and A in SNPs rs6691378 and rs10399805 increased and tended to increase the risk of developing cervical pre-cancerous lesions (OR: 1.66, 95% CI: 1.11–2.49 and OR: 1.48, 95% CI: 0.98–2.25, respectively; Table 3). They also increased the risk of developing invasive cervical cancer (OR: 1.51, 95% CI: 1.08–2.12 and OR: 1.60, 95% CI: 1.14–2.25, respectively; Table 3).

**Table 3:** Allele distributions of the single nucleotide polymorphisms of the *chitinase 3-like 1* gene in patients with invasive cancer and pre-cancerous lesions of the uterine cervix and normal controls.

Variables	Normal controls (n = 310)	Pre-cancerous lesions (n = 61)	Invasive cancer(n = 99)	*P* (power)^a^	OR (95% CI)^b^	OR (95% CI)^c^
rs6691378						
G^d^	450	75	126	0.008^a^	1.00	1.00
A	170	47	72	(0.8)	1.66 (1.11–2.49)	1.51 (1.08–2.12)
rs10399805						
G^d^	462	81	128	0.012^a^	1.00	1.00
A	158	41	70	(0.8)	1.48 (0.98–2.25)	1.60 (1.14–2.25)
rs4950928						
C^d^	508	103	174	0.140	1.00	1.00
G	112	19	24		0.84 (0.49–1.42)	0.63 (0.39–1.00)
rs880633						
T^d^	410	91	128	0.144	1.00	1.00
C	210	31	70		0.66 (0.43–1.03)	1.07 (0.76–1.49)

Statistical analysis: Chi-square test.

a *p*<0.05; the sample size was estimated with power (1-β), 0. 80 and α, 0.05 and the powers were calculated when the comparisons among the subgroups reached statistical significances (*p*<0.05) with an adequate sample size using WinPepi software, version 10.0.

b Comparison between patients with pre-cancerous lesions and normal controls.

c Comparison between patients with cervical cancer and normal controls.

d Used as a reference to evaluate the odds ratio.

Abbreviations: OR, odds ratio; 95% CI, 95% confidence interval.

### Haplotypes of *chitinase 3-like 1* SNPs based on Taiwanese women and their involvement in cervical cancer

**Figure 1:**
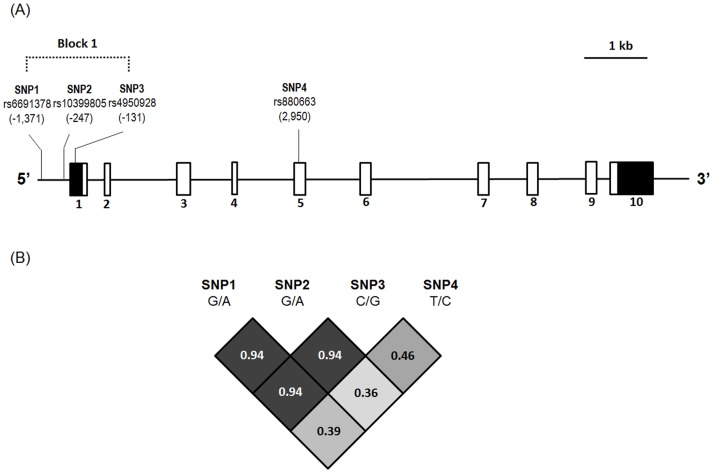
*Chitinase 3-like 1* (*CHI3L1*) gene, locations of the genotyped variants, and their pairwise linkage disequilibrium (LD) patterns. (A) Schematic presentation of the *CHI3L1* (gene ID: 1116) indicates the locations of the analyzed variants (rs6691378, rs10399805, rs4950928, and rs880663). The black and white boxes indicate the un-translated and coding regions, respectively. The exon number labeled below the exons and variant locations begins with the translation start site. (B) The one observed haploblock that the pairwise LD measured is D'labeled and colored in gray scale using the population data from Han Chinese in Beijing, China (CHB) in the HapMap 3.

Based on the locations of the analyzed variants (rs6691378, rs10399805, rs4950928 and rs880663), the *CHI3L1* gene, locations of the genotyped variants, and their pairwise linkage disequilibrium patterns were plotted (Figure 1). Because the mutant homozygous AA and AA in *CHI3L1* SNPs rs6691378 and rs10399805 increased the risk of developing cervical pre-cancerous lesions or invasive cancer, haplotypes containing them (AACC and AACT) were regarded as a risk subgroup, while other haplotypes (i.e., GGCC, GGCT, GGGC, GGGT, GACC, GACT, AGCC, and AGCT) were regarded as a control subgroup. Individuals with haplotypes AACC and AACT had an increased risk of developing cervical neoplasia (*p* = 0.002). When the cervical neoplasia group was further subdivided into subgroups of invasive cancer and pre-cancerous lesions, significant differences existed in the distributions of *CHI3L1* haplotypes among the women with cervical invasive cancer and pre-cancerous lesions and the controls (*p* = 0.01). The women with the AACC or AACT haplotypes had a higher risk of developing invasive cervical cancer (OR: 1.60, 95% CI: 1.13–2.26; Table 4).

**Table 4:** Haplotype distributions of the *chitinase 3-like 1* (*CHI3L1*) gene in the patients with invasive cancer or pre-cancerous lesions of the uterine cervix and normal controls.

*CHI3L3* haplotypes^a^	Control women	Patients with pre-cancerous lesions	Patients with invasive cancer	*P* (power)^b^	OR and 95% CI^c^	OR and 95% CI^d^
AACC and AACT	153	41	68	0.01 (0.8)	1.55 (1.02–2.35)	1.60 (1.13–2.26)
Others	467	81	130		1.00 (reference)	1.00 (reference)

a Haplotypes containing AACC and AACT were regarded as a risk subgroup; other haplotypes (GGCC, GGCT, GGGC, GGGT, GACC, GACT, AGCC and AGCT) were regarded as a control subgroup.

b *p*<0.05; the sample size was estimated with power (1-β), 0.80 and α, 0.05 and the powers were calculated when the comparisons among the subgroups reached statistical significances (*p*<0.05) with an adequate sample size using WinPepi software, version 10.0.

c Comparison between patients with pre-cancerous lesions and normal controls.

d Comparison between patients with cervical cancer and normal controls.

Abbreviations: OR, odds ratio; 95% CI, 95% confidence interval.

### Association of *chitinase 3-like 1* gene polymorphisms and haplotypes with clinicopathologic variables of cervical cancer, cancer recurrence, and patient survival

There were no associations between *CHI3L1* gene polymorphisms and clinicopathologic variables of cervical cancer, cancer recurrence, and patient survival. When the *CHI3L1* haplotypes were included in the analysis, they were related to the clinical stage (*p* = 0.040, OR: 2.31, 95% CI: 0.95–5.72; ≥ stage II vs. stage I; Table 5), tumor diameter (*p* = 0.054; OR: 2.19, 95% CI: 0.90–5.43; >4 cm vs. ≤4 cm) and vaginal invasion (*p* = 0.024; OR: 2.66, 95% CI: 0.98–6.83; invasion vs. no invasion). Moreover, significant associations became even more obvious among the *CHI3L1* risk haplotypes and clinical stage (*p* = 0.009; OR: 3.29, 95% CI: 1.18–9.63; ≥ stage II vs. stage I; Table 5), tumor diameter (*p* = 0.029; OR: 2.75, 95% CI: 0.99–8.04; >4 cm vs. ≤4 cm), and vaginal invasion (*p* = 0.004; OR: 4.10, 95% CI: 1.40–11.62; invasion vs. no invasion) in SCC specimens, but not in adenocarcinoma tissues.

**Table 5:** Association of haplotype distribution of *chitinase 3-like 1* polymorphisms with clinicopathologic characteristics of the patients with uterine cervical cancer.

Variables^a^	Haplotypes AACC or AACT vs. control haplotypes^c^	*p* and odds ratio (95/% confidence interval)	Haplotypes AACC or AACT vs. control haplotypes^c^ in SCC tissues	*p* and odds ratio (95/% confidence interval) for SCC
Clinical stage		0.04		0.009
stage I^b^	12 104	1.00	8 94	1.00
≥stage II	16 60	2.31 (0. 95–5.72)	14 50	3.29 (1.18–9.63)
Pathologic type squamous cell		0.187	unavailable	unavailable
carcinoma^b^	22 144	1.00		
adenocarcinoma	6 20	1.96 (0.58–5.81)		
Cell grading		0.354		0.084
well (grade 1)^b^	8 34	1.00	8 26	1.00
moderate & poor (grades 2/3)	20 130	0.65 (0.25–1.87)	14 118	2.59 (0.84–7.42)
Invasion depth of cervical stroma		0.354		0.476
≤10 mm^b^	18 90	1.00	14 80	1.00
>10 mm	10 74	0.68 (0.26–1.66)	8 64	0.71 (0.24–1.96)
Tumor diameter^b^		0.054		0.029
≤4 cm	12 102	1.00	8 88	1.00
>4 cm	16 62	2.19 (0.90–5.43)	14 56	2.75 (0.99–8.04)
Parametrium		0.637		0.261
no invasion^b^	20 124	1.00	14 108	1.00
invasion	8 40	1.24 (0.44–3.22)	8 36	1.71 (0.57–4.80)
Vagina		0.024		0.004
no invasion^b^	18 134	1.00	16 108	1.00
invasion	10 28	2.66 (0.98–6.83)	6 36	4.10 (1.40–11.62)
Pelvic lymph node		0.637		0.819
no metastasis^b^	20 124	1.00	16 108	1.00
metastasis	8 40	1.24 (0.44–3.22)	6 36	1.13 (0.33–3.32)

Statistical analyses: Chi-square test.

a Some clinicopathologic data could not be collected from the patients with cervical cancer due to incomplete medical charts or records.

b As a reference.

c Haplotypes containing AACC and AACT were regarded as a risk subgroup; other haplotypes (GGCC, GGCT, GGGC, GGGT, GACC, GACT, AGCC and AGCT) were regarded as a control subgroup for comparison.

Abbreviation: SCC, squamous cell carcinoma.

Cervical cancer patients with *CHI3L1* risk haplotypes tended to have poor survival event (logistic regression model; *p* = 0.078; OR:2.99, 95% CI: 0.89–10.08; Table 6). However, cell grading 2/3 (*p* = 0.049; OR: 5.47, 95% CI: 1.01–29.78) and positive pelvic lymph node metastasis (*p* = 0.002; OR: 5.58, 95% CI: 1.92–12.63) were independent predictive factors for patient survival event. The five-year survival rate was 66.1% for the patients with the *CHI3L1* risk haplotypes, and this increased to 88.1% for those with other haplotypes. However, it could not reach a statistical significance (*p* = 0.21; OR: 1.68, 95% CI: 0.73–3.85; Table 7). Moreover, *CHI3L1* haplotypes also had a tendency to be related to a higher probability of recurrence event (*p* = 0.081; OR: 3.07, 95% CI: 0.87–10.81; Table 6). Cell grading 2/3 (*p* = 0.015; OR: 8.73, 95% CI: 1.52–50.29) and positive pelvic lymph node metastasis (*p* = 0.001; OR: 6.52, 95% CI: 2.25–18.93) were independent predictive factors for cancer recurrence event. In the cervical cancer patients with SCC, *CHI3L1* risk haplotypes increased the risk of recurrence event (*p* = 0.011; OR: 7.50, 95% CI: 1.60–35.16) and tended to increase the risk of poor survival event (*p* = 0.051; OR: 4.36, 95% CI: 0.99–19.14), in logistic regression model. Deep invasion depth and ≥stage II also increased the risk of recurrence event (*p* = 0.04; OR: 4.61, 95% CI: 1.08–19.74 and *p* = 0.047; OR: 6.17, 95% CI: 1.03–37.04, respectively). Positive pelvic lymph node metastasis increased the risk of recurrence event (*p* = 0.012; OR: 4.49, 95% CI: 1.40–14.46) and tended to increase the risk of poor survival event (*p* = 0.058; OR: 3.10, 95% CI: 0.96–10.00).

**Table 6:** Influence of *chitinase 3-like 1* (*CHI3L1*) haplotypes and clinicopathologic parameters on cancer recurrence event and survival event of the patients with uterine cervical cancer.

Variables	Recurrence event		Survival event	
	*p* value	OR & 95%CI^b^	*p* value	OR & 95%CI^b^
*CHI3L1* risk haplotypes^a^	0.081	3.07 (0.87–10.81)	0.078	2.99 (0.89–10.08)
Cell grading 2/3	0.015	8.73 (1.52–50.29)	0.049	5.47 (1.01–29.78)
Positive pelvic lymph node metastasis	0.001	6.52 (2.25–18.93)	0.002	5.58 (1.92–16.23)

Variables in patients with squamous cell carcinoma	Recurrence event		Survival event	
	*p* value	OR & 95%CI^b^	*p* value	OR & 95%CI^b^
*CHI3L1*risk haplotypes^a^	0.011	7.50 (1.60–35.16)	0.051	4.36 (0.99–19.14)
≥stage II	0.047	6.17 (1.03–37.04)	insignificant	
Deep stromal invasion depth	0.04	4.61 (1.08–19.74)	insignificant	
Positive pelvic lymph node metastasis	0.012	4.49 (1.40–14.46)	0.058	3.10 (0.96–10.00)

Statistical analysis: logistic regression model.

a Haplotypes containing AACC and AACT were regarded as a risk subgroup; other haplotypes (GGCC, GGCT, GGGC, GGGT, GACC, GACT, AGCC and AGCT) were regarded as a control subgroup for comparison.

b OR and 95% CI, odds ratio and 95% confidence interval for risk haplotypes AACC and AACT and poor clinicopathologic characteristics, compared to their respective controls.

**Table 7:** Univariate and multivariate analyses for the correlation of clinicopathologic variables and *CHI3L1* risk haplotype expressions with overall survival of the patients with uterine cervical cancer.

	5-year survival rate (%)^b^	Hazard ratio^c^	95% confidence interval^c^	*p* value
**Univariate analysis**				
Stage^a^				
I	91.0	1	Reference	0.02
others	77.8	2.57	1.12–5.91	
Pathologic type^a^				
squamous cell	86.1	1	Reference	
carcinoma				0.18
adenocarcinoma	66.8	1.84	0.74–4.59	
Depth of stromal				
invasion^a^				0.0024
≤1/2 depth	90.0	1	Reference	
>1/2 depth	75.1	3.28	1.44–7.46	
Tumor diameter^a^				
≤4 cm	92.6	1	Reference	0.0011
>4 cm	73.4	3.55	1.56–8.06	
Cell grading^a^ well	94.1	1	Reference	0.0091
moderate and poor	80.2	5.49	1.29–23.26	
Parametrialinvasion^a^				
no invasion	88.8	1	Reference	0.016
invasion	74.6	2.40	1.14–5.08	
Vagina invasion^a^				
no invasion	85.6	1	Reference	0.20
invasion	80.5	1.64	0.75–3.57	
Pelvic lymph node metastasis^a^				
negative	90.2	1	Reference	0.0068
positive	67.0	2.72	1.27–5.81	
*CHI3L1* risk haplotypes expression				
negative	88.1	1	Reference	0.21
positive	66.1	1.68	0.73–3.85	
**Multivariate analysis**				
Cell grading				
well (grade 1)^a^	94.1	1	Reference	0.037
moderate & poor (grades 2/3)	80.2	5.59	1.11–28.57	

Statistical analysis: Kaplan-Meier model and multivariate and univariate Cox regression models.

a Some clinicopathologic data could not be collected from the patients with cervical cancer due to incomplete medical charts or records.

b Analyzed by the Kaplan-Meier model.

c Based on overall survival analyzed by multivariate and univariate Cox regression models.

Abbreviation: *CHI3L1*, *chitinase 3-like 1*.

Only cell grading was found to be an independent predictor for overall survival in multivariate analysis (*p* = 0.037, hazard ratio: 5.59, 95% CI: 1.11–28.57; Table 7). However, *CHI3L1* risk haplotypes, depth of stromal invasion, cell grading and pelvic lymph node metastasis were independent predictive factors for the probability of recurrence (Table 8). In multivariate analysis for the probability of recurrence and overall survival of the patients with SCC, *CHI3L1* risk haplotypes exhibited a significant association with the probability of recurrence (*p* = 0.002; hazard ratio: 6.21, 95% CI: 1.90–20.41) and a marginal association with overall survival (*p* = 0.051; hazard ratio: 3.76, 95% CI: 0.99–14.29) among the clinicopathologic parameters and *CHI3L1* haplotypes, using the Cox proportional hazards model (Table 8). However, these findings were not demonstrated in the patients with adenocarcinoma.

**Table 8:** Influence of *chitinase 3-like 1* (*CHI3L1*) haplotypes and clinicopathologic parameters on cancer recurrence probability and overall survival of the patients with uterine cervical cancer.

Variables	Recurrence		Overall survival	
	*p* value	OR & 95%CI^b^	*p* value	OR & 95%CI^b^
*CHI3L1* risk haplotypes^a^	0.009	3.57 (1.37–9.35)	0.174	1.99 (0.74–5.35)
Depth of stromal invasion	0.036	3.47 (1.09–11.11)	insignificant	
Cell grading 2/3	0.042	5.21 (1.06–25.64)	0.037	5.59 (1.11–28.57)
Positive pelvic lymph node metastasis	0.002	5.26 (1.85–15.15)	insignificant	

Variables in patients with squamous cell carcinoma	Recurrence		Overall survival	
	*p* value	OR & 95%CI^b^	*p* value	OR & 95%CI^b^
*CHI3L1* risk haplotypes^a^	0.002	6.21 (1.90–20.41)	0.051	3.76 (0.99–14.29)
Deep stromal invasion depth	0.027	5.00 (1.20–20.83)	insignificant	

Statistical analysis: multivariate Cox regression model.

a Haplotypes containing AACC and AACT were regarded as a risk subgroup; other haplotypes (GGCC, GGCT, GGGC, GGGT, GACC, GACT, AGCC and AGCT) were regarded as a control subgroup for comparison.

b OR and 95% CI, odds ratio and 95% confidence interval for risk haplotypes AACC and AACT and poor clinicopathologic characteristics, compared to their respective controls.

## Discussion

To the best of our knowledge, the present study is the first to show a significant association between *CHI3L1* gene polymorphisms and susceptibility to uterine cervical cancer. Both mutant homozygous genotypes AA and AA in the promoter regions −1371 and −247 of *CHI3L1* SNPs rs6691378 and rs10399805 not only increased the susceptibility to pre-cancerous lesions but also invasive cancer of the uterine cervix even after controlling for age. No such differences were observed in rs4950928 (−131, promoter region) and rs880633 (+2950, exon 5). Several studies on *CHI3L1* SNPs have documented that the genetic variations of *CHI3L1* affect circulating YKL-40 levels, both in healthy adults and in patients with diseases [17]–[20]. Kajergarred et al. and Thomsen et al. demonstrated the regulatory effects of some promoter SNPs, including rs6691378, rs10399805, and rs4950928, on plasma YKL-40 levels in the Danish general population [21], [22]. The minor allele of rs10399805 has been reported to enhance the binding of CCAAT enhancer-binding protein (C/EBFα) to the gene promoter and increase *CHI3L1* transcription, consequently increasing plasma YKL-40 levels [23], [24]. In contrast, Zheng et al. did not show any significant association between SNP rs10399805 and YKL-40 levels in a Chinese population [17]. Different study populations may affect the results. Verlaan et al. revealed that rs10399805 and rs4950928 modulate *CHI3L1* transcription and promoter polymorphisms in *CHI3L1* and are associated with asthma [25]. Most of the susceptibility genes for common diseases do not play a vital role in predisposing to the disease, but act as response modifiers to internal or external environmental factors [26], [27].

The YKL-40 protein is encoded by the *CHI3L1*, and SNPs in the *CHI3L1* promoter have been associated with elevated serum YKL-40 levels [20], [28], and differential gene expression [28] and transcript levels [29]. YKL-40 has been reported to initiate the mitogen-activated protein kinase (MAPK) and phosphoinoside-3-kinase (PI3K) signaling pathways, which lead to increased cell proliferation in connective-tissue cells [30]. The murine homologue of YKL-40 is a breast regression protein of 39 kDa (BRP-39) which has been described to be expressed in cancer cells, and YKL-40/BRP-39 has been demonstrated to play a role in cell proliferation, survival, and tissue remodeling [30]–[32]. Serum YKL-40 can be secreted from fully activated macrophages associated with the tumor and can be produced by tumor cells themselves or by non-malignant cells such as activated neutrophils and fibroblasts, chondrocytes, and synovial cells [32]–[34]. Tumor cells have been reported to express and produce YKL-40 in breast and colon cancer [35], while tumor-associated macrophages, but not tumor cells, have been reported to secrete YKL-40 in lung cancer [36]. Mitsuhashi et al. found that pre-treatment serum levels of YKL-40 are elevated in cervical cancer, even in the early stages [15]. Because *CHI3L1* SNPs influence YKL-40 expression, they may subsequently influence the development of diseases such as cervical cancer.

The MAFs in rs6691378, rs10399805, and rs4950928 of *CHI3L1* promoters in the Taiwanese women in the current study (27.4%, 25.5%, and 18.1%, respectively) are similar to those reported in the Han Chinese in Beijing, China (HCB, 25.6%, 25.6%, and 17.1%, respectively), which are based on the National Center for Biotechnology Information (NCBI) SNP database (dbSNP). The MAF in rs880633 (33.9%) in exon 5 of the *CHI3L1* gene is also similar to that in HCB (35.6%), which is also based on the dbSNP. Furthermore, the mutant allele A in SNP rs6691378 increased the risk of developing cervical pre-cancerous lesions and invasive cancer. Although the mutant allele A in SNP rs10399805 also increased susceptibility to cervical cancer, its effect on the development of pre-cancerous lesions was not as strong as that of A in SNP rs6691378. In order to apply the haplotypes, pairwise linkage disequilibrium patterns were established for these SNPs, and the results revealed that rs6691378 has a strong linkage disequilibrium with rs10399805 (D′ = 0.94). The mutant homozygous AA as well as AA in *CHI3L1* SNPs rs6691378 and rs10399805 were associated with cervical carcinogenesis. Thus, *CHI3L1* haplotypes may be used for further correlation with the development and clinicopathologic variables of cervical cancer and patient prognosis.

Not only the allele distribution of *CHI3L1* SNPs but also *CHI3L1* risk haplotypes, AACC and AACT, correlated with the susceptibility to cervical pre-cancerous lesions and invasive cancer in the present study. There are few existing studies that have reported the relationships of *CHI3L1* haplotypes with diseases. Zhao et al. revealed significant associations of three SNPs in the promoter region of the *CHI3L1* gene (rs6691378, −1371; rs10399805, −247 and rs4950928, −131) with schizophrenia [28]. They further constructed these three SNPs as *CHI3L1* haplotypes and found that the *CHI3L1* haplotypes implicated in schizophrenia susceptibility were associated with altered expression levels of the gene. Genetic variations that change the expression of *CHI3L1* may influence some key processes that are *CHI3L1*-dosage dependent. One is the AKT-mediated signal pathway through PI3K-dependent phosphorylation [30]. This AKT pathway has been associated with cell survival [37], [38] and may regulate cytokine-induced cellular responses [39]. Nonetheless, Zheng et al. could not demonstrate any relationship between *CHI3L1* common haplotypes and coronary artery disease or its severity [17].

Mitsuhashi et al. demonstrated that serum YKL-40 level is the best biomarker for uterine cervical cancer compared to the SCC antigens, CA-125, CA19-9, and C-reactive protein [15]. Pre-treatment YKL-40 levels were significantly correlated with FIGO stage and with relapse or persistent disease status, while correlations with nodal status and tumor size were marginal in cervical adenocarcinoma. Cox regression analysis showed that an elevated YKL-40 level is associated with relapse or persistent disease in cervical adenocarcinoma [15]. YKL-40 has been suggested to play a role in cell proliferation, differentiation, and to protect cells from apoptotic signals, and act on extracellular tissue remodeling [30], [40]. Reported as a stimulator of angiogenesis in tumors, YKL-40 is posited to be involved in cancer metastasis [41], [42]. The present study postulates that *CHI3L1* SNPs or haplotypes affect the expression of YKL-40. Analyzing the relationship among *CHI3L1* SNPs or haplotypes, clinicopathologic variables, cancer recurrence, and patient survival, we could not demonstrate any significant association between *CHI3L1* SNPs and these characteristics. However, cervical cancer patients with the *CHI3L1* risk haplotypes AACC or AACT had significant associations with clinical stage and vaginal invasion, while the association with tumor size was marginal. In cervical cancer patients with SCC, these relationships were more obvious. Logistic regression model revealed that the patients with the risk haplotypes tend to have higher recurrence event and poorer survival event. The other independent factors were cell grading and pelvic lymph node metastasis. In the patients with SCC, *CHI3L1* risk haplotypes more obviously increased the risk of recurrence event and poor survival event. In multivariate Cox proportional hazard model, there was a significant association between *CHI3L1* risk haplotypes and the probability of recurrence, and a marginal association between risk haplotypes and overall survival in the patients with cervical SCC. However, these findings were not seen in the patients with adenocarcinoma, implying that *CHI3L1* risk haplotypes are particularly applicable for predicting the prognosis in patients with SCC, especially in terms of recurrence.

In conclusion, the *CHI3L1* SNPs rs6691378 and rs10399805 and *CHI3L1* haplotypes were correlated with the development of pre-cancerous lesions and invasive cancer of the uterine cervix. Cervical cancer patients with the *CHI3L1* haplotypes AACC or AACT had poor clinicopathologic characteristics and a poor prognosis. These risk haplotypes were associated with a higher probability of recurrence, especially for those with SCC.
